# Pressure-Dependent Diffusion of PEG-Functionalized Gold Nanoparticles Probed by X‑ray Photon Correlation Spectroscopy

**DOI:** 10.1021/acs.jpcb.6c03752

**Published:** 2026-08-29

**Authors:** Nele N. Striker, Florian Schulz, Christina Krywka, Claudia Goy, Svenja C. Hövelmann, Niels C. Giesselmann, Fabian Westermeier, Frédéric Caupin, Michael Paulus, Felix Lehmkühler

**Affiliations:** † 28332 Deutsches Elektronen-Synchrotron DESY, 22603 Hamburg, Germany; ‡ Center for Hybrid Nanostructures, Universität Hamburg, 22761 Hamburg, Germany; § Helmholtz-Zentrum Hereon, 28338 Institute for Materials Physics, Max-Planck-Str. 1, 21502 Geesthacht, Germany; ∥ Institute of Experimental and Applied Physics, Kiel University, Leibnizstraße 19, 24118 Kiel, Germany; ⊥ Institut Lumière Matière, Université Lyon 1, CNRS, Institut Universitaire de France, F-69622 Villeurbanne, France; # Fakultät Physik/DELTA, TU Dortmund, 44221 Dortmund, Germany

## Abstract

Dynamic properties of nanoparticles under high pressure are poorly understood due to experimental challenges. Here, we use X-ray photon correlation spectroscopy (XPCS) to investigate the diffusion dynamics of polyethylene glycol (PEG)-functionalized gold nanoparticles dispersed in water at pressures up to 6 kbar. Complementary small-angle X-ray scattering reveals no measurable pressure-induced changes in the gold core size, confirming structural stability across the studied pressure range. XPCS measurements show a systematic slowing down of nanoparticle dynamics with increasing pressure, with relaxation rates retaining the expected *q* ^2^-dependence characteristic of Brownian motion. The extracted diffusion coefficients decrease continuously with pressure and align with predictions based on the pressure-dependent viscosity of water and the Stokes–Einstein relation, assuming a constant hydrodynamic particle size. Within experimental uncertainty, no additional contribution from pressure-induced ligand-shell modifications is observed in these dilute dispersions.

## Graphical abstract

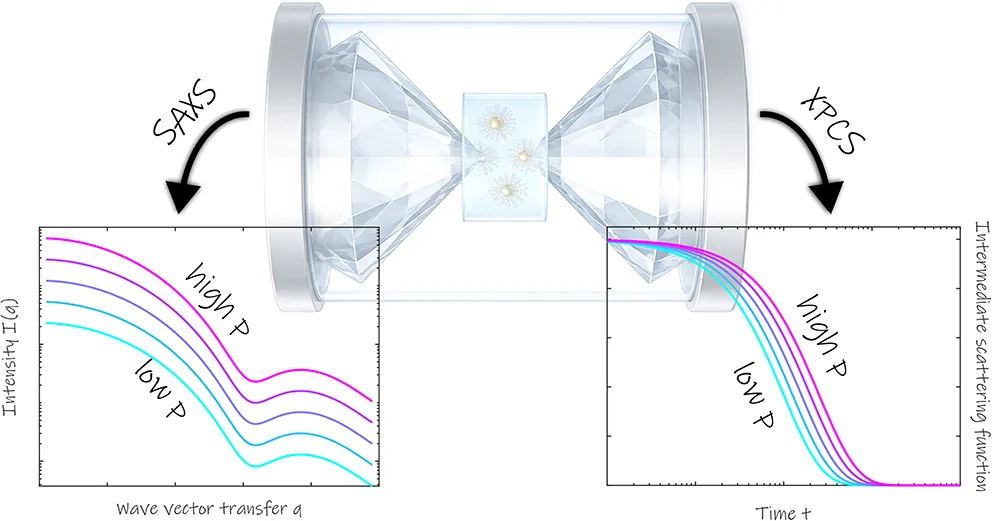

## Introduction

Understanding the dynamic behavior of nanoparticles in solution is crucial for advancing fields such as nanotechnology, soft matter physics, and biophysics. These systems exhibit complex transport phenomena that govern processes like self-assembly, drug delivery, and catalytic efficiency. Typically, in situ dynamics of dispersed nanoparticles are accessed via correlation techniques such as dynamic light scattering (DLS)^1–3^ or its X-ray analogue, X-ray photon correlation spectroscopy (XPCS).^4–6^ However, probing such dynamics under extreme conditions—particularly at high hydrostatic pressure—remains a significant experimental challenge.^7–12^ While ambient-pressure studies have provided valuable insights into nanoparticle diffusion,^13–20^ the behavior of these systems under pressures reaching gigapascals (GPa) is far less explored.

The structure and function of soft and biological matter under high hydrostatic pressures are active areas of research,^21–26^ yet a critical gap persists in our understanding of their dynamics. This gap stems largely from the technical difficulties of high-pressure experiments, which require specialized equipment capable of withstanding extreme forces while maintaining precise control over experimental conditions.^12,27–29^

One key property influencing nanoparticle dynamics is the viscosity of the surrounding solvent, which can change dramatically under high pressure (e.g., water^30–34^ ). Various methods have been developed to measure the viscosity under pressure (see, for instance, ref 35 for a review on the international formulation for the viscosity of water). However, these methods are more challenging than traditional rheometry at ambient pressure. Moreover, the interplay between pressure-induced changes in solvent structure and nanoparticle motion adds another layer of complexity.^36^ XPCS offers a unique solution to these challenges.^6,29,37^ It provides direct access to the temporal and spatial correlations of nanoparticle motion, enabling the extraction of diffusion coefficients and other dynamic parameters with high precision. Its ability to probe nanoscale dynamics in situ makes it particularly well suited for high-pressure studies, where sample environments are often confined and optically inaccessible.

Recent advances in XPCS have dramatically expanded its capabilities. The newest generation of synchrotron radiation and free-electron laser (FEL) sources has enabled access to shorter time scales, from femto- to nanosecond dynamics at FELs using split-pulse methods^38–42^ to (sub)­microsecond dynamics at storage rings and FELs via detector improvements.^43–52^ The increased degree of coherence at shorter wavelengths has recently enabled XPCS studies under high pressures, despite the challenges posed by thick windows. Examples include dynamics of metallic glasses in diamond anvil cells,^53–55^ transitions in amorphous ices,^56^ and cluster dynamics in supercritical CO_2_ using split-pulse XPCS at FELs.^41^ In soft matter, high-pressure XPCS has been applied to pressure-induced liquid–liquid transitions in aqueous lysozyme solutions,^57^ colloidal diffusion up to 1.4 kbar,^58^ coil-to-globule transitions and gelation of temperature-responsive polymers,^59^ and dynamic transitions in hydrated protein powders.^60^

Here, we apply XPCS to study the diffusion of gold nanoparticles as a function of pressure. The particles are functionalized with polyethylene glycol (PEG)-based ligands and dispersed in water. Previously, we investigated their diffusion dynamics as a function of particle size and temperature, finding good agreement with the Stokes–Einstein relation.^52^ However, this relationship may break down under high hydrostatic pressures. These particles exhibit a transition from repulsive to attractive interactions above 2000 bar,^61^ which can ultimately lead to pressure-induced supercrystal formation.^62^ This transition is governed by modulation of the PEG-based ligands’ thickness and conformation. Compressibility of PEG ligands has been widely studied by atomic force microscopy, where the ligands’ response on the AFM tip is recorded,^63–66^ but little is known about the behavior of PEG ligands at high hydrostatic pressures. In this work, we address this question via tracking particle dynamics up to 6 kbar (600 MPa) using XPCS. By extracting diffusion coefficients as a function of both particle size and pressure, we aim to disentangle whether the change in particle–particle interactions via ligand response is a pure pressure effect or emerges only in dense systems.

## Methods

This section provides an overview of the XPCS technique, the gold nanoparticle samples, and experimental details.

### X-ray Photon Correlation Spectroscopy

XPCS provides access to sample dynamics via correlations of intensities *I*(*q*,*t*) at time *t* and the modulus of the wave vector transfer $q=\left|q\right|=\frac{4\pi }{\lambda }\sin\left(\theta /2\right)$ , with wavelength λ and scattering angle θ. Typically, it studies the correlation function

1 $$g_{2}\left(q,\Delta t\right)=\frac{\left\langle I\left(q,t\right)I\left(q,t+\Delta t\right)\right\rangle }{\langle I\left(q,t\right)\rangle^{2}}$$

that correlates the scattered intensity *I*(*q*,*t*) at a time *t* with that at time *t* + Δ*t*.^5,6,67^ The averaging is performed over time *t* and detector pixels belonging to the same *q*-bin. Using the Siegert relation, the *g*_2_ function is related to the correlation function of the field, known as the intermediate scattering function *f*(*q*,Δ*t*), as

2 $$g_{2}\left(q,\Delta t\right)=1+\beta |f\left(q,\Delta t\right){|}^{2}$$

Here, β is the speckle contrast that contains information on the coherence of the X-rays. XPCS is thus extending dynamic light scattering by being able to access shorter length scales $d\approx \frac{2\pi }{q}$ , and with fewer limitations, e.g., it allows probing turbid samples and is not affected by multiple scattering.^68,69^

In the case of diffusive dynamics, *g*_2_ can be described by

3 $$g_{2}\left(q,\Delta t\right)=1+\beta \exp\left(-2\Gamma \Delta t\right)$$

Here, Γ≡1/τ is the relaxation rate and τ is the relaxation time, which is given by

4 $$\Gamma =Dq^{2}=\frac{k_{B}T}{6\pi \eta R}q^{2}$$

with the Stokes–Einstein diffusion coefficient *D*, temperature *T*, Boltzmann’s constant *k*_B_, viscosity η, and particle radius *R*. Deviations from such a diffusive behavior may lead to a variation of the factor 6. For instance, due to hydrodynamic boundary conditions, it can vary from 4 (full slip) to 6 (no slip).^70,71^

### Samples

The gold nanoparticles (AuNP) were synthesized using the seeded growth method,^72^ resulting in different generations of particles with increasing size. The final particles were coated with ligand shells of α-methoxypoly­(ethylene glycol)-ω-(11-mercaptoundecanoate) (PEGMUA).^73^ PEGMUA ligands with 2 kDa were used, resulting in a shell thickness of *s* ≈ 9.6 nm. An overview of the different particle sizes studied is given in Table 1. For some samples, dilutions were studied as well as the stock sample. Note that the volume fraction was always in the range ϕ∼10^–4^ to avoid particle–particle interactions.

**Table 1:** Sample Overview Showing the Radius of the Gold Core *r*_core_ and the Volume Fraction *ϕ* of the Stock Solution^a^

sample	*r*_core_ (nm)	ϕ (×10^–4^)
AuNP1	34.8	2.6
AuNP2	36.7	2.5
AuNP3	43.4	1.9
AuNP4	47.6	3.7

a The gold core radii were obtained by modeling the scattered intensity with a spherical form factor (see SI).

### Experiment

The XPCS experiment was performed at beamline P10 of PETRA III at DESY (Hamburg, Germany). An ultrasmall-angle X-ray scattering (USAXS) geometry with a sample–detector distance of 21.2 m was used. The detector was a DECTRIS EIGER 500k with a pixel size of 75 × 75 μm^2^ and a maximum frame rate of 9 kHz. The beam size was set to 100 × 100 μm^2^ at the sample position, defined by slits. A photon energy of 13 keV was chosen to reduce the X-ray absorption through the 2 mm thick diamond windows of the pressure cell. This cell allows experiments on liquids up to 7 kbar hydrostatic pressure, similar to the cell presented by Krywka et al.^27^ The samples were filled in dedicated sample holders with a thickness of 1.5 mm. After the sample holder was positioned in the pressure chamber, XPCS runs were taken at different spots. The pressure was adjusted using a screw pump and measured with a gauge (Nova Swiss). The measurements started at 100 bar, followed by a measurement at 500 bar. Afterward, the pressure was typically increased in 500 bar steps. The pressure was increased up to 6 kbar, and further runs were taken while releasing pressure in 1000 bar steps. At each pressure, two XPCS measurements were performed. Each XPCS measurement had an exposure time of 0.12 ms per frame and consisted of 10 batches with 6000 frames each, resulting in 60000 measured speckle patterns. To avoid radiation damage, each batch was measured on a different spot on the sample holder. All data has been modeled using error-weighted (χ^2^) fits to the data.

## Results and Discussion

First, we discuss the pressure dependence of the static scattering of the samples. The scattered intensities were obtained for each sample at each pressure by averaging the speckle patterns over the azimuthal angle ω. Since all samples had a low volume fraction of ϕ < 10^–3^, the scattered intensity can be modeled by the single-particle form factor. Selected scattered intensities of AuNP3 are shown in Figure 1(a,b) for increasing and decreasing pressure. Data from all samples are shown in the SI. The intensity profiles show the same trend for all measured pressures; the intensity decreases as a function of *q* and has a minimum at *q* ≈ 0.1 nm^–1^. Due to the low electron density difference between the PEGMUA ligands and the surrounding water, the scattering is dominated by the gold cores.^61,74,75^ As no change is observed for increasing or decreasing pressure, this suggests that the gold core does not change with pressure. This matches previous small-angle X-ray scattering (SAXS) studies at high pressures.^36,61,62,76^

**Figure 1:**
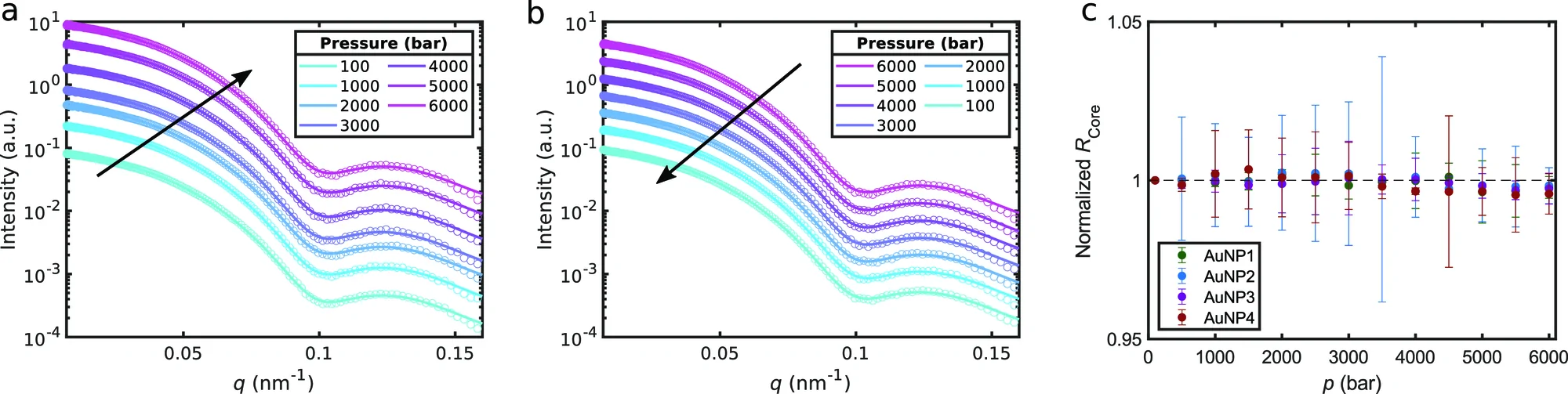
Azimuthally averaged scattered intensities *I*(*q*) for sample AuNP3 (a) during pressure increase and (b) during pressure release. The lines represent fits of a form factor model of disperse spheres. The data are vertically offset for clarity. (c) Normalized core radii *r*_core_(*p*) /*r*_core_(100 bar) as a function of pressure obtained from the fits to the data shown in (a) and (b).

For all samples, the core radius *r*_core_ was determined at each pressure by fitting a form factor model of disperse spheres to the intensity profiles. The size dispersity was modeled by a Schulz-Zimm distribution that provides a good description for colloidal sphere. Details can be found in ref.^77^ The results are given in Table 1. For all samples, the dispersity was found to be around 11%. In order to compare all samples, the normalized *r*_core_/*r*_core_(100 bar) is shown in Figure 1 (c) as a function of pressure *p*. This indicates that the core radius remains constant in the pressure range of 100–6000 bar, as expected thanks to the low compressibility of gold.^78^

The dynamic properties of the AuNPs were investigated using XPCS. The intermediate scattering functions were obtained from the *g*_2_-functions using eq 2. The speckle contrast for this experiment was β ≈ 0.04 (see SI). The intermediate scattering functions of AuNP3 are shown in Figure 2(a) for different *q*-values at *p* = 100 bar. They decrease from 1 to 0 with increasing delay time Δ*t*. Especially at the larger *q*-values, the correlation functions could not be fully resolved within the accessible detector time window (see the discussion in ref 52); only the last part of the decay of |*f*(*q*,*t*)|^2^ could be resolved. Notably, the *g*_2_ functions could be modeled well using a single exponential decay (eq 3) for all samples and pressures.

**Figure 2:**
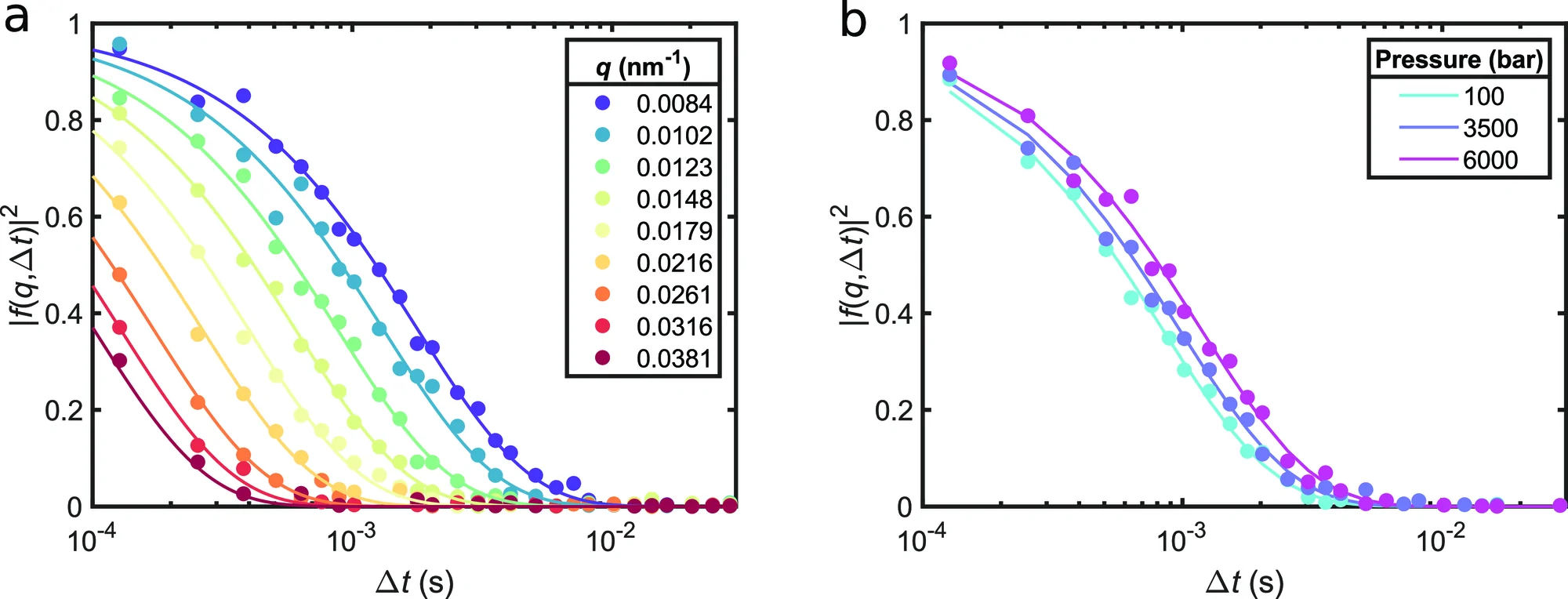
XPCS results for sample AuNP3. (a) Intermediate scattering function (ISF) as a function of *q* at *p* = 100 bar. (b) ISF at three selected increasing pressures for *q* = 0.0123 nm^–1^. The lines are fits to the data.

The role of pressure on the dynamics is highlighted in Figure 2(b). Here, intermediate scattering functions of AuNP3 are shown for a fixed *q* value at three selected pressures. With increasing pressure, |*f*(*q*,*t*)|^2^ shifts to slightly longer time scales, indicating a slowing down of the dynamics.

To quantify and model this slowdown of the dynamics, a more detailed analysis of the relaxation times has been performed. These relaxation times τ were determined from the intermediate scattering functions for the various *q*-values and pressures by fitting eq 3 to the data, as discussed in the previous paragraphs. The resulting values of τ are shown in Figure 3(a) for AuNP3 for various *q*-values at three selected pressures. For all pressures, τ decreases with increasing *q*. For *q* > 0.03 nm^–1^, the dynamics were too fast to resolve and the intermediate scattering functions could not be measured with sufficient statistics (see Figure 2). Thus, in the following analysis, relaxation times τ are only considered up to *q* = 0.03 nm^–1^.

**Figure 3:**
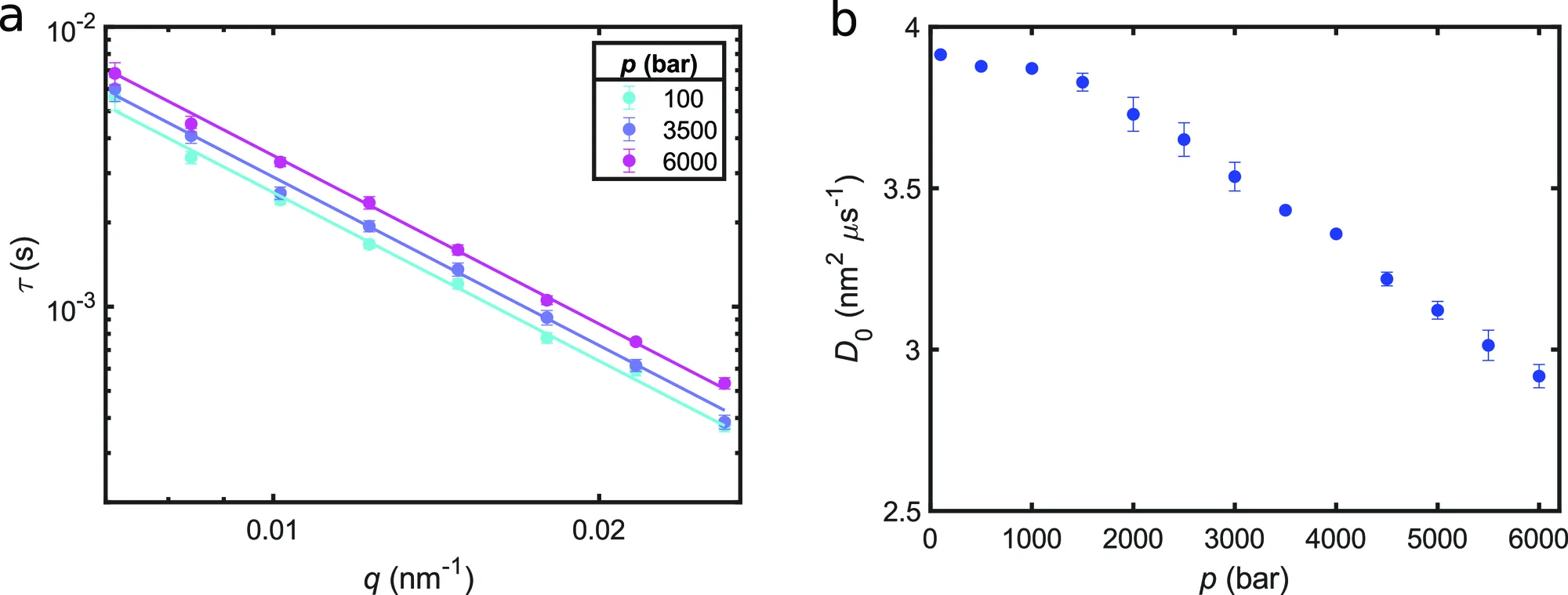
(a) Relaxation time τ as a function of *q* for three selected pressures for sample AuNP3. (b) Diffusion coefficients as a function of pressure for sample AuNP3.

First, a function of type τ = 1/(*D*·*q* ^*n*^) was fitted to the data to determine the diffusion coefficient *D*. Since the fits showed an exponent *n* ≈ 2 for all pressures—as expected for diffusive dynamics—*n* was set to 2, and the fit had only one free parameter *D*. A weighted average of *D* was performed over all XPCS measurements for each sample and is shown in Figure 3(b) for AuNP3. Diffusion coefficients of all samples are shown in the SI. The weights are given by the errors on *D* obtained from the τ vs *q* fit. *D* decreases with increasing pressure *p*.

In order to focus on the pressure dependence alone, the radius-independent, normalized diffusion coefficient *D*/*D*_100bar_ is studied and shown in Figure 4(a). The error bars are given by the error-weighted standard deviation of all measurements at different radii. The data follow the expectation from the literature, assuming only a pressure-induced change of viscosity (black dashed line).^32,33^ In addition, the behavior of the additional change of radius with pressure as reported by Schroer et al.^61^ up to 4 kbar is shown as well as a solid blue line. This line is just within the error bars of the data, but systematically above the average value. Therefore, the match between experimental data and the constant radius model suggests that the particles’ radius does not change with pressure. In particular, the deviation from the best-case extrapolation assuming only the viscosity change and no further radius change beyond 4 kbar supports this conclusion. In addition, Figure 4(b) shows the extracted viscosity values from the data shown in Figure 4(a).

**Figure 4:**
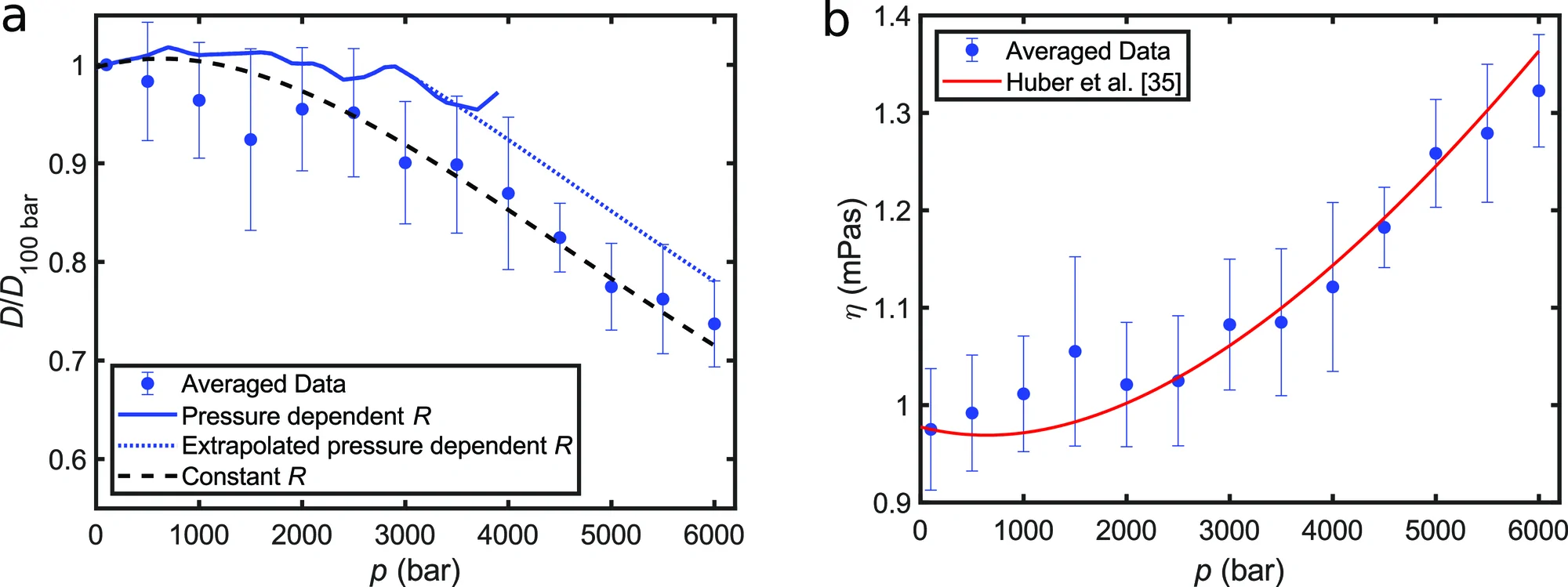
(a) Weighted average *D*/*D*_100 bar_ as a function of pressure. Pressures measured for single samples only are given by triangles. The black dashed line shows the expected diffusion coefficient for a constant hydrodynamic radius, assuming pressure-dependent water viscosity.^32,33,35^ The solid blue line shows the results using a radius variation as reported in ref 61 for pressures up to 4000 bar. The exact radius values have been used, resulting in the wavy curve. The dashed blue line represents an extrapolation of that data to higher pressures. (b) Extracted viscosity values using eq 4. The solid red line represents literature values.^35^

The absence of a measurable deviation from the viscosity-controlled Stokes–Einstein behavior suggests that pressure-induced ligand-shell modifications do not substantially affect the hydrodynamic drag of isolated nanoparticles in dilute solution. In concentrated dispersions, however, even subtle changes in PEG ligand conformation or compressibility may significantly modify interparticle interaction potentials, steric repulsion, and effective attraction ranges. Consequently, collective phenomena such as aggregation or supercrystal formation^61,62^ can become highly pressure-sensitive even when the single-particle diffusion coefficient remains dominated by the pressure dependence of the solvent viscosity.

## Conclusions

In this work, we demonstrated the application of XPCS to investigate nanoparticle diffusion under high hydrostatic pressures of up to 6 kbar. PEGMUA-functionalized gold nanoparticles dispersed in water were studied by using a dedicated high-pressure sample environment at beamline P10 of PETRA III.

The static SAXS data revealed no measurable pressure-induced changes in the gold core size over the investigated pressure range. At the same time, XPCS measurements showed a clear slowing of the nanoparticle dynamics with increasing pressure. The extracted relaxation rates followed the expected diffusive *q* ^2^ dependence, indicating that the particle motion remains Brownian throughout the investigated pressure regime.

The measured pressure dependence of the diffusion coefficients is consistent with the increase in water viscosity under pressure and agrees well with predictions based on the Stokes–Einstein relation assuming a constant hydrodynamic radius. Within the experimental uncertainty, no additional contribution from a pressure-induced change of the effective particle size was observed for the dilute systems studied here. This suggests that previously reported pressure-induced modifications of PEG ligand conformations predominantly affect dense systems with stronger interparticle interactions rather than isolated nanoparticles in dilute dispersion.

These results establish high-pressure XPCS as a powerful tool for probing nanoscale dynamics in extreme sample environments at modern X-ray sources. Compared to experiments with visible light,^32^ it allows extension to molecular length scales and thus probes length-scale-dependent dynamic properties such as viscosity even in aqueous systems. The approach opens new opportunities for studying pressure-dependent transport phenomena, interaction potentials, and phase transitions in soft matter, including concentrated colloids, pressure-induced aggregation,^61^ soft matter phase transitions,^59,62^ and biomolecular dynamics.^57,60^

## Supplementary Material



## Notes

SAXS data and diffusion coefficients of all samples studied; raw *g*_2_ data for sample AuNP3 and a static colloidal glass (PDF)

## References

[1] Berne, B. J. ; Pecora, P. R. Dynamic Light Scattering: With Applications to Chemistry, Biology, and Physics; Dover Publications Inc., 2000; p 384.

[2] Stetefeld J., McKenna S. A., Patel T. R. (2016). Dynamic
light scattering: a practical
guide and applications in biomedical sciences. Biophys. Rev..

[3] Filippov S. K., Khusnutdinov R., Murmiliuk A., Inam W., Zakharova L. Y., Zhang H., Khutoryanskiy V. V. (2023). Dynamic light scattering and transmission
electron microscopy in drug delivery: a roadmap for correct characterization
of nanoparticles and interpretation of results. Mater. Horiz..

[4] Sandy A. R., Zhang Q., Lurio L. B. (2018). Hard X-Ray Photon Correlation Spectroscopy
Methods for Materials Studies. Annu. Rev. Mater.
Res..

[5] Madsen, A. ; Fluerasu, A. ; Ruta, B. Synchrotron Light Sources and Free-Electron Lasers; Springer International Publishing, 2020; pp 1989–2018.

[6] Lehmkühler F., Roseker W., Grübel G. (2021). From Femtoseconds
to Hours–Measuring
Dynamics over 18 Orders of Magnitude with Coherent X-rays. Appl. Sci..

[7] Wu H., Wang Z., Fan H. (2014). Stress-Induced Nanoparticle Crystallization. J. Am. Chem. Soc..

[8] Saccone F. D., Ferrari S., Errandonea D., Grinblat F., Bilovol V., Agouram S. (2015). Cobalt ferrite nanoparticles under high pressure. J. Appl. Phys..

[9] Kulkarni C. V., Yaghmur A., Steinhart M., Kriechbaum M., Rappolt M. (2016). Effects of High Pressure on Internally
Self-Assembled
Lipid Nanoparticles: A Synchrotron Small-Angle X-ray Scattering (SAXS)
Study. Langmuir.

[10] Zhu H., Nagaoka Y., Hills-Kimball K., Tan R., Yu L., Fang Y., Wang K., Li R., Wang Z., Chen O. (2017). Pressure-Enabled Synthesis of Hetero-Dimers
and Hetero-Rods through
Intraparticle Coalescence and Interparticle Fusion of Quantum-Dot-Au
Satellite Nanocrystals. J. Am. Chem. Soc..

[11] Lehmkühler F., Schroer M. A., Markmann V., Frenzel L., Möller J., Lange H., Grübel G., Schulz F. (2019). Kinetics of pressure-induced
nanocrystal superlattice formation. Phys. Chem.
Chem. Phys..

[12] Meng L., Vu T. V., Criscenti L. J., Ho T. A., Qin Y., Fan H. (2023). Theoretical and Experimental
Advances in High-Pressure Behaviors
of Nanoparticles. Chem. Rev..

[13] Caronna C., Chushkin Y., Madsen A., Cupane A. (2008). Dynamics of Nanoparticles
in a Supercooled Liquid. Phys. Rev. Lett..

[14] Katayama K., Nomura H., Ogata H., Eitoku T. (2009). Diffusion coefficients
for nanoparticles under flow and stop-flow conditions. Phys. Chem. Chem. Phys..

[15] Conrad H., Lehmkühler F., Fischer B., Westermeier F., Schroer M. A., Chushkin Y., Gutt C., Sprung M., Grübel G. (2015). Correlated heterogeneous dynamics in glass-forming
polymers. Phys. Rev. E.

[16] Zhang C., Jin Z., Zeng B., Wang W., Palui G., Mattoussi H. (2020). Characterizing
the Brownian Diffusion of Nanocolloids and Molecular Solutions: Diffusion-Ordered
NMR Spectroscopy vs Dynamic Light Scattering. J. Phys. Chem. B.

[17] Liu W., Zheng B., Yin X., Yu X., Zhang Y., Wiegart L., Fluerasu A., Armstrong B. L., Veith G. M., Bhatia S. R. (2021). XPCS Microrheology and Rheology of
Sterically Stabilized Nanoparticle Dispersions in Aprotic Solvents. ACS Appl. Mater. Interfaces.

[18] Berkowicz S., Perakis F. (2021). Exploring the validity
of the Stokes-Einstein relation
in supercooled water using nanomolecular probes. Phys. Chem. Chem. Phys..

[19] Rodriguez-Loya J., Lerma M., Gardea-Torresdey J. L. (2024). Dynamic Light Scattering and Its
Application to Control Nanoparticle Aggregation in Colloidal Systems:
A Review. Micromachines.

[20] Otto F., Dallari F., Westermeier F., Wieland D. C. F., Parak W. J., Lehmkühler F., Schulz F. (2024). The dynamics of PEG-coated nanoparticles
in concentrated protein solutions up to the molecular crowding range. Aggregate.

[21] Mozhaev V. V., Heremans K., Frank J., Masson P., Balny C. (1996). High pressure
effects on protein structure and function. Proteins.

[22] Tiwari A., Butler I. S., Kozinski J. A. (2009). A Brief
Overview of the Effect of
High Pressures on the Vibrational Spectra of Biomaterials. Appl. Spectrosc. Rev..

[23] Möller J., Schroer M. A., Erlkamp M., Grobelny S., Paulus M., Tiemeyer S., Wirkert F., Tolan M., Winter R. (2012). The Effect
of Ionic Strength, Temperature, and Pressure on the Interaction Potential
of Dense Protein Solutions: From Nonlinear Pressure Response to Protein
Crystallization. Biophys. J..

[24] Brooks N. J., Seddon J. M. (2014). High Pressure X-ray
Studies of Lipid Membranes and
Lipid. Phase Trans. Z. Phys. Chem..

[25] Meersman F., McMillan P. F. (2014). High hydrostatic
pressure: a probing tool and a necessary
parameter in biophysical chemistry. Chem. Commun..

[26] Papadakis C. M., Niebuur B.-J., Schulte A. (2024). Thermoresponsive
Polymers under Pressure
with a Focus on Poly­(N-isopropylacrylamide) (PNIPAM). Langmuir.

[27] Krywka C., Sternemann C., Paulus M., Tolan M., Royer C., Winter R. (2008). Effect of Osmolytes on Pressure-Induced
Unfolding of
Proteins: A High-Pressure SAXS Study. ChemPhysChem.

[28] Urban V. S., Heller W. T., Katsaras J., Bras W. (2021). Soft Matter Sample
Environments for Time-Resolved Small Angle Neutron Scattering Experiments:
A Review. Appl. Sci..

[29] Muhunthan P., Li H., Vignat G. (2024). A versatile pressure-cell design for studying
ultrafast molecular-dynamics in supercritical fluids using coherent
multi-pulse x-ray scattering. Rev. Sci. Instrum..

[30] Wonham J. (1967). Effect of
Pressure on the Viscosity of Water. Nature.

[31] Först P., Werner F., Delgado A. (2000). The viscosity
of water at high pressures
- especially at subzero degrees centigrade. Rheol. Acta.

[32] Eichler J., Stefanski J., Roca J. M., Daniel I., Issenmann B., Valeriani C., Caupin F. (2025). Shear and Bulk Viscosities of Water
up to 1.6 GPa and Anomaly in the Structural Relaxation Time. Phys. Rev. Lett..

[33] Singh L. P., Issenmann B., Caupin F. (2017). Pressure dependence of viscosity
in supercooled water and a unified approach for thermodynamic and
dynamic anomalies of water. Proc. Natl. Acad.
Sci. U.S.A..

[34] Mussa A., Berthelard R., Caupin F., Issenmann B. (2023). Viscosity
and Stokes-Einstein relation in deeply supercooled water under pressure. J. Chem. Phys..

[35] Huber M. L., Perkins R. A., Laesecke A., Friend D. G., Sengers J. V., Assael M. J., Metaxa I. N., Vogel E., Mareš R., Miyagawa K. (2009). New International Formulation
for the Viscosity of
H2O. J. Phys. Chem. Ref. Data.

[36] Martín-Sánchez C., Sánchez-Iglesias A., Barreda-Argüeso J. A., Polian A., Liz-Marzán L.
M., Rodríguez F. (2023). Behavior of
Au Nanoparticles under Pressure Observed by In Situ Small-Angle X-ray
Scattering. ACS Nano.

[37] Zhou Z., Zhang M., Cui C., Wei L., Li S., Guo Z., Xu Y., Tian F., Li X., Jiang H., Tai R. (2025). Strategies to perform and optimize
x-ray photon correlation spectroscopy
experiments. Phys. Scr..

[38] Roseker W., Hruszkewycz S. O., Lehmkühler F. (2018). Towards ultrafast dynamics
with split-pulse X-ray photon correlation spectroscopy at free electron
laser sources. Nat. Commun..

[39] Shinohara Y., Osaka T., Inoue I., Iwashita T., Dmowski W., Ryu C. W., Sarathchandran Y., Egami T. (2020). Split-pulse X-ray photon
correlation spectroscopy with seeded X-rays from X-ray laser to study
atomic-level dynamics. Nat. Commun..

[40] Sun Y., Dunne M., Fuoss P., Robert A., Zhu D., Osaka T., Yabashi M., Sutton M. (2020). Realizing split-pulse
x-ray photon correlation spectroscopy to measure ultrafast dynamics
in complex matter. Phys. Rev. Res..

[41] Majumdar A., Li H., Muhunthan P., Späh A., Song S., Sun Y., Chollet M., Sokaras D., Zhu D., Ihme M. (2024). Direct observation
of ultrafast cluster dynamics in supercritical carbon dioxide using
X-ray Photon Correlation Spectroscopy. Nat.
Commun..

[42] Goy C., Dallari F., Roseker W. (2025). Speckle contrast from
the split-and-delay unit with seeded X-ray pulses of the MID instrument
at European XFEL. J. Phys. Conf. Ser..

[43] Zhang Q., Dufresne E. M., Narayanan S., Maj P., Koziol A., Szczygiel R., Grybos P., Sutton M., Sandy A. R. (2018). Sub-microsecond-resolved
multi-speckle X-ray photon correlation spectroscopy with a pixel array
detector. J. Synchrotron Radiat..

[44] Lehmkühler F., Dallari F., Jain A. (2020). Emergence of anomalous
dynamics in soft matter probed at the European XFEL. Proc. Natl. Acad. Sci. U.S.A..

[45] Dallari F., Jain A., Sikorski M. (2021). Microsecond hydrodynamic
interactions in dense colloidal dispersions probed at the European
XFEL. IUCrJ.

[46] Reiser M., Girelli A., Ragulskaya A. (2022). Resolving molecular
diffusion and aggregation of antibody proteins with megahertz X-ray
free-electron laser pulses. Nat. Commun..

[47] Girelli A., Bin M., Filianina M. (2025). Coherent X-rays reveal anomalous molecular
diffusion and cage effects in crowded protein solutions. Nat. Commun..

[48] Anthuparambil N. D., Dargasz M., Timmermann S. (2026). Lipoprotein diffusion
in dense yolk plasma is governed by softness, hydrodynamics, and caging:
Insights from MHz-XPCS. Proc. Natl. Acad. Sci.
U.S.A..

[49] Jo W., Westermeier F., Rysov R. (2021). Nanosecond X-ray photon
correlation spectroscopy using pulse time structure of a storage-ring
source. IUCrJ.

[50] Jo W., Stern S., Westermeier F., Rysov R., Riepp M., Schmehr J., Lange J., Becker J., Sprung M., Laurus T., Graafsma H., Lokteva I., Grübel G., Roseker W. (2023). Single and multi-pulse
based X-ray photon correlation
spectroscopy. Opt. Express.

[51] Chushkin Y., Correa J., Ignatenko A., Pennicard D., Lange S., Fridman S., Karl S., Senfftleben B., Lehmkühler F., Westermeier F., Graafsma H., Cammarata M. (2025). Achieving
nanosecond time resolution with a two-dimensional X-ray detector. J. Synchrotron Radiat..

[52] Striker N. N., Schulz F., Goy C., Correa J., Simancas A., Dallari F., Marzi D., Gautam R. P., Arauz-Moreno C., Bauer R. P., Chèvremont W., Cammarata M., Graafsma H., Caupin F., Lehmkühler F. (2026). XPCS at the
Microsecond Frontier: Diffusion of PEGylated Nanoparticles in Water. Mater. Adv..

[53] Cornet A., Garbarino G., Zontone F. (2023). Denser glasses relax
faster: Enhanced atomic mobility and anomalous particle displacement
under in-situ high pressure compression of metallic glasses. Acta Mater..

[54] Zhang X., Lou H., Ruta B., Chushkin Y., Zontone F., Li S., Xu D., Liang T., Zeng Z., Mao H.-k., Zeng Q. (2023). Pressure-induced
nonmonotonic cross-over of steady relaxation dynamics in a metallic
glass. Proc. Natl. Acad. Sci. U.S.A..

[55] Cornet A., Ronca A., Shen J., Zontone F., Chushkin Y., Cammarata M., Garbarino G., Sprung M., Westermeier F., Deschamps T., Ruta B. (2024). High-pressure X-ray photon correlation
spectroscopy at fourth-generation synchrotron sources. J. Synchrotron Radiat..

[56] Karina A., Li H., Eklund T. (2025). Multicomponent
dynamics in amorphous ice studied
using X-ray photon correlation spectroscopy at elevated pressure and
cryogenic temperatures. Commun. Chem..

[57] Moron M., Al-Masoodi A., Lovato C., Reiser M., Randolph L., Surmeier G., Bolle J., Westermeier F., Sprung M., Winter R., Paulus M., Gutt C. (2022). Gelation Dynamics
upon Pressure-Induced Liquid-Liquid Phase Separation in a Water-Lysozyme
Solution. J. Phys. Chem. B.

[58] Chèvremont W., Zinn T., Narayanan T. (2024). Improvement
of ultra-small-angle
XPCS with the Extremely Brilliant Source. J.
Synchrotron Radiat..

[59] Striker N. N., Krywka C., Goy C., Hövelmann S., Giesselmann N. C., Schulz F., Lokteva I., Westermeier F., Caupin F., Paulus M., Lehmkühler F. (2025). Phase behavior
of Silica-PNIPAm nanogels under highhydrostatic pressure. J. Appl. Crystallogr..

[60] hlfeldt M., Bin M., Girelli A., Andronis I., Karina A., Das Anthuparambil N., Berner F., Eklund T., Kraft L. E., Leonau A., Westermeier F., Sprung M., Gutt C., Amann-Winkel K., Perakis F. (2026). X-ray photon correlation spectroscopy of hydrated lysozyme
at elevated pressures. Phys. Chem. Chem. Phys..

[61] Schroer M. A., Schulz F., Lehmkühler F., Möller J., Smith A. J., Lange H., Vossmeyer T., Grübel G. (2016). Tuning the Interaction of Nanoparticles from Repulsive
to Attractive by Pressure. J. Phys. Chem. C.

[62] Schroer M. A., Lehmkühler F., Möller J., Lange H., Grübel G., Schulz F. (2018). Pressure-Stimulated Supercrystal Formation in Nanoparticle
Suspensions. J. Phys. Chem. Lett..

[63] Nnebe I. M., Schneider J. W. (2006). A Tapping-Mode
AFM Study of the Compression of Grafted
Poly­(ethylene glycol) Chains. Macromolecules.

[64] del
Pino P., Yang F., Pelaz B., Zhang Q., Kantner K., Hartmann R., Martinez de Baroja N., Gallego M., Möller M., Manshian B. B., Soenen S. J., Riedel R., Hampp N., Parak W. J. (2016). Basic Physicochemical Properties of Polyethylene Glycol
Coated Gold Nanoparticles that Determine Their Interaction with Cells. Angew. Chem., Int. Ed..

[65] Lemoine P., Dooley C., Morelli A., Harrison E., Dixon D. (2022). AFM study
of organic ligand packing on gold for nanoparticle drug delivery applications. Appl. Surf. Sci..

[66] Latag G. V., Tahara H., Katase A., Maeda S., Liu Y., Kakuta Y., Teramoto T., Mori T., Hayashi T. (2026). AFM-Based
Single-Molecule Force Spectroscopy of PEG–Anti-PEG Antibody
Interactions. ACS Appl. Bio Mater..

[67] Perakis F., Gutt C. (2020). Towards molecular movies
with X-ray photon correlation spectroscopy. Phys. Chem. Chem. Phys..

[68] Semeraro E. F., Möller J., Narayanan T. (2018). Multiple-scattering effects in SAXS
and XPCS measurements in the ultra-small-angle region. J. Appl. Crystallogr..

[69] Jo W., Rysov R., Westermeier F., Walther M., Müller L., Philippi-Kobs A., Riepp M., Marotzke S., Lokteva I., Sprung M., Grübel G., Roseker W. (2022). Demonstration of 3D
photon correlation spectroscopy in the hard X-ray regime. Opt. Lett..

[70] Hodgdon J. A., Stillinger F. H. (1993). Stokes-Einstein violation in glass-forming liquids. Phys. Rev. E.

[71] Schmidt J. R., Skinner J. L. (2003). Hydrodynamic boundary conditions, the Stokes-Einstein
law, and long-time tails in the Brownian limit. J. Chem. Phys..

[72] Bastús N. G., Comenge J., Puntes V. (2011). Kinetically Controlled Seeded Growth
Synthesis of Citrate-Stabilized Gold Nanoparticles of up to 200 nm:
Size Focusing versus Ostwald Ripening. Langmuir.

[73] Schulz F., Vossmeyer T., Bastús N. G., Weller H. (2013). Effect of the Spacer
Structure on the Stability of Gold Nanoparticles Functionalized with
Monodentate Thiolated Poly­(ethylene glycol) Ligands. Langmuir.

[74] Larson-Smith K., Pozzo D. C. (2011). Scalable synthesis of self-assembling nanoparticle
clusters based on controlled steric interactions. Soft Matter.

[75] Iranpour
Anaraki N., Liebi M., Ong Q., Blanchet C., Maurya A. K., Stellacci F., Salentinig S., Wick P., Neels A. (2021). In-situ Investigations on Gold Nanoparticles
Stabilization Mechanisms in Biological Environments Containing HSA. Adv. Funct. Mater..

[76] Schulz F., Möller J., Lehmkühler F., Smith A. J., Vossmeyer T., Lange H., Grübel G., Schroer M. A. (2018). Structure and Stability
of PEG- and Mixed PEG-Layer-Coated Nanoparticles at High Particle
Concentrations Studied In Situ by Small-Angle X-Ray Scattering. Part. Part. Syst. Char..

[77] Westermeier F., Fischer B., Roseker W., Grübel G., Nägele G., Heinen M. (2012). Structure and short-time
dynamics
in concentrated suspensions of charged colloids. J. Chem. Phys..

[78] Dubrovinsky L., Dubrovinskaia N., Crichton W. A., Mikhaylushkin A. S., Simak S. I., Abrikosov I. A., de Almeida J. S., Ahuja R., Luo W., Johansson B. (2007). Noblest of
All Metals Is Structurally Unstable at High Pressure. Phys. Rev. Lett..

