# Supplementary material for: Pressure-Dependent Diffusion of PEG-Functionalized Gold Nanoparticles Probed by X‑ray Photon Correlation Spectroscopy
Source: J Phys Chem B. 2026 Aug 29;130(36):9268–75. doi: 10.1021/acs.jpcb.6c03752 (PMC13573565; doi:10.1021/acs.jpcb.6c03752)
Supplement: Supplementary file 1 [file jp6c03752_si_001.pdf]

Supplementary Information: Pressure-Dependent Diffusion of PEG-Functionalized Gold Nanoparticles Probed by X-Ray Photon Correlation Spectroscopy

Nele N. Striker[1], Florian Schulz[2], Christina Krywka[3], Claudia Goy[1], Svenja C. Hövelmann[1,4], Niels C. Giesselmann[1], Fabian Westermeier[1], Frédéric Caupin[5], Michael Paulus[6], and Felix Lehmkühler*[1]

[1]Deutsches Elektronen-Synchrotron DESY, 22603 Hamburg, Germany.
[2]Center for Hybrid Nanostructures, Universität Hamburg, 22761 Hamburg, Germany.
[3]Helmholtz-Zentrum Hereon, Institute for Materials Physics, Max-Planck-Str. 1, 21502 Geesthacht, Germany.
[4]Institute of Experimental and Applied Physics, Kiel University, Leibnizstraße 19, 24118 Kiel, Germany.
[5]Institut Lumière Matière, Université Lyon 1, CNRS, Institut Universitaire de France, F-69622 Villeurbanne, France.
[6]Fakultät Physik/DELTA, TU Dortmund, 44221 Dortmund, Germany.

*Email: felix.lehmkuehler@desy.de

Scattered Intensities

The scattered intensities are shown in Fig. S1 for all samples at 100 bar. The intensity decreases with increasing q and the first minimum of the form factor is visible for all samples.

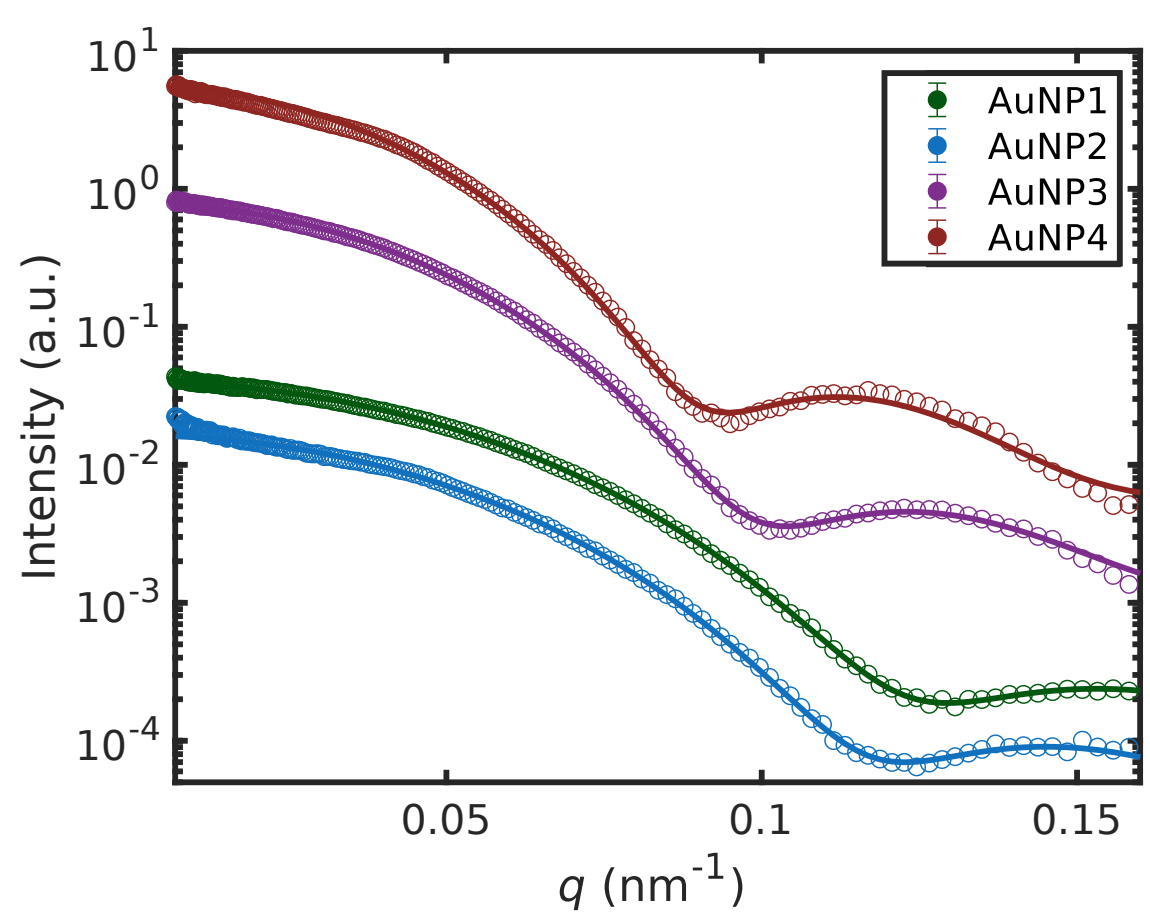

Figure S1: Azimuthally averaged scattered intensities as a function of q for all samples at 100 bar. The circles correspond to the data points, the lines correspond to the respective fits. The intensity profiles have been shifted vertically for clarity.

Correlation functions

Raw g_2 data and fits are shown in Fig. S2 for sample AuNP3. Data from a static glass sample are shown as well as a reference for obtaining speckle contrast values. For the q-range under study, the speckle contras was constant around 4 %, as indicated by the error bars in Fig. S2.

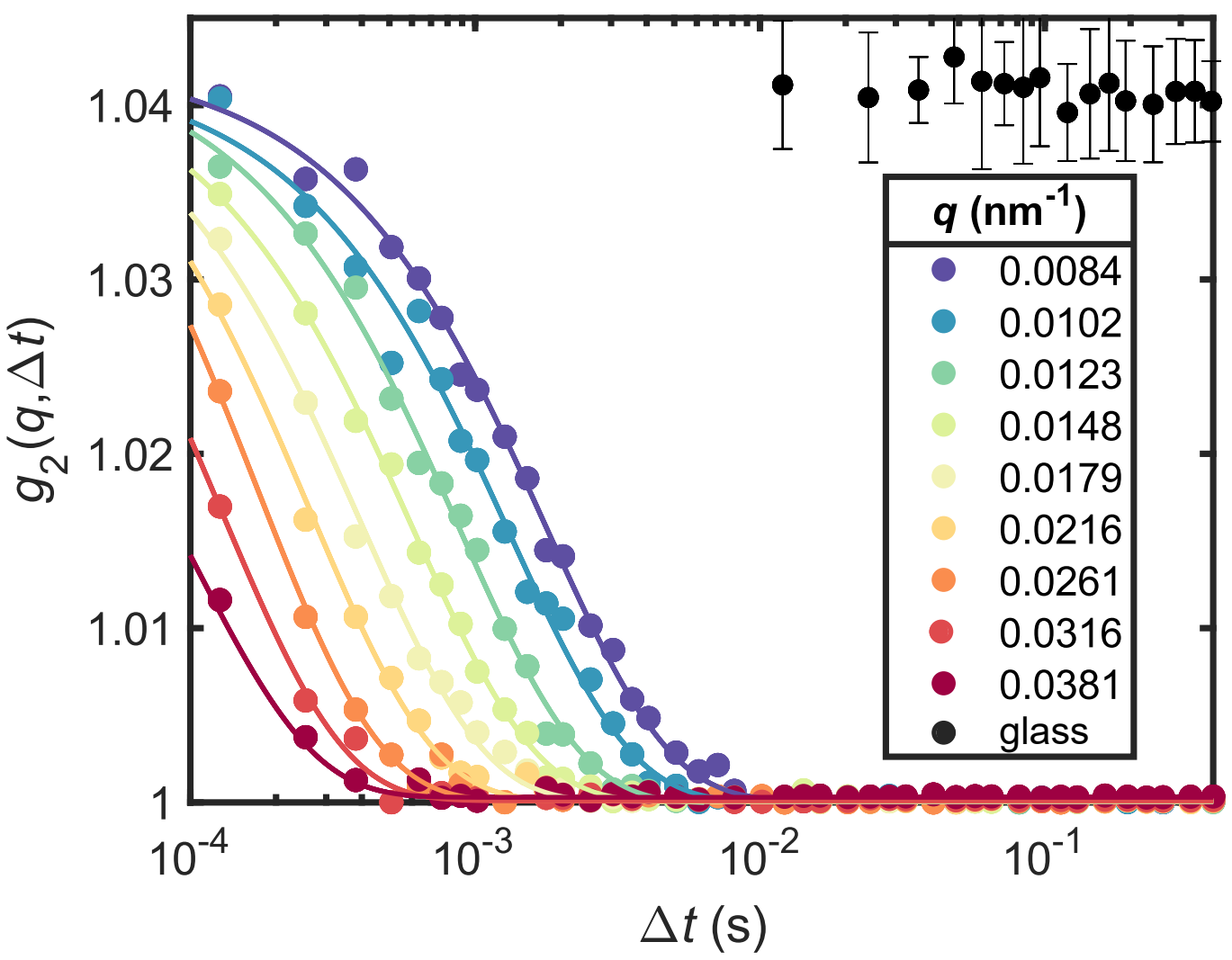

Figure S2: Raw g_2 data for sample AuNP3. The black dots are g_2 data taken from a static glass sample.

Diffusion Coefficients

This section shows supplementary data for the diffusion coefficients. Fig. S3 shows the extracted diffusion coefficients for all measured samples. Additionally to the stock solution, AuNP1 and AuNP4 were measured as a 1:1 and 1:2 dilution with water, respectively. With their experimental uncertainty, the diffusion coefficients are the same for stock and dilute solution under the same conditions.

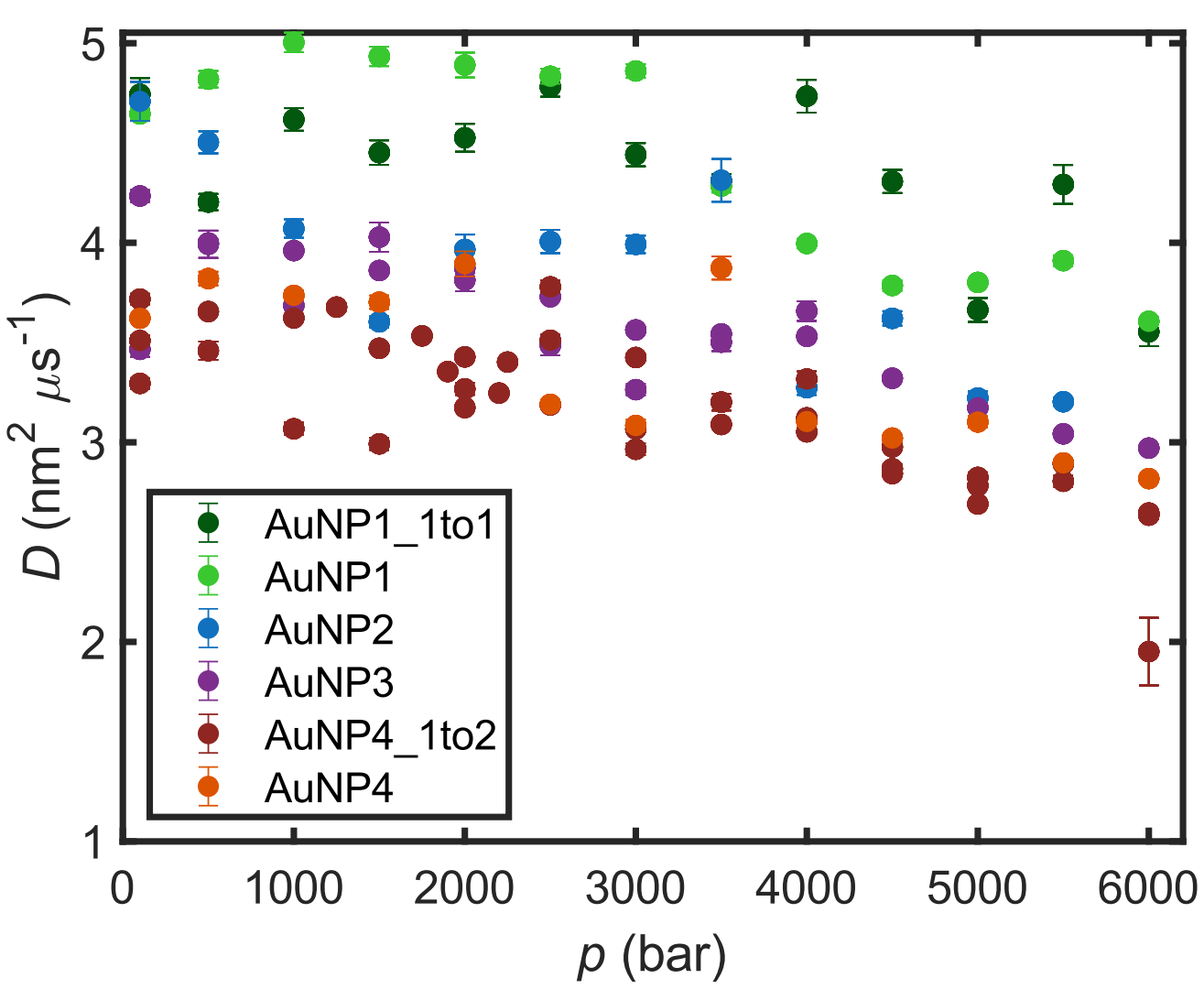

Figure S3: Diffusion coefficients D as a function of pressure for all measured samples. The additions '_1to1' and '_1to2' denote the dilutions of the respective stock samples.

The diffusion coefficients normalized to the diffusion coefficient at $p = 100$ bar are shown in Fig. S4 for all samples. Additionally, the weighted average, as shown in Fig. 4 in the main manuscript, is displayed.

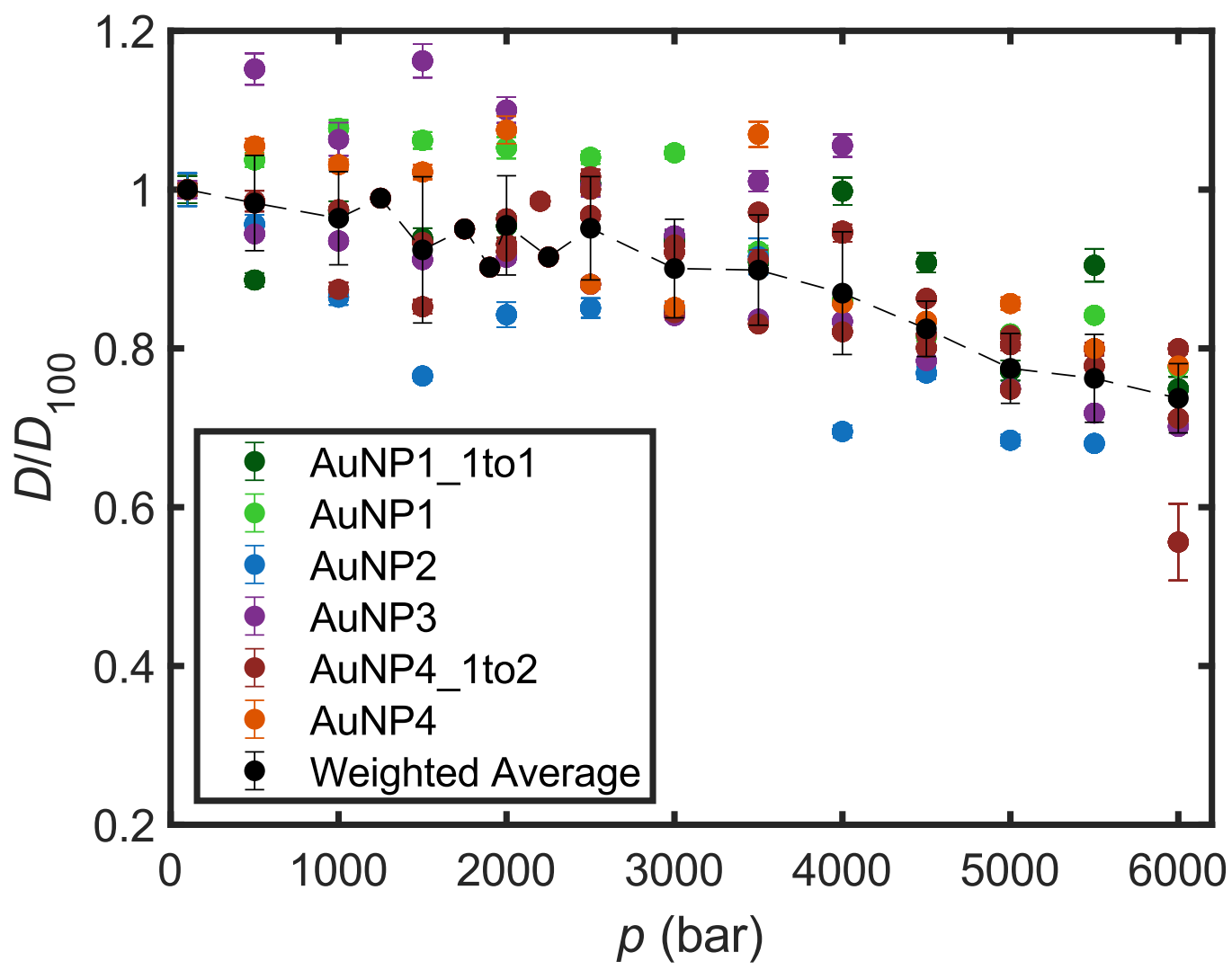

Figure S4: Normalized diffusion coefficients D/D_{100} as a function of pressure for all measured samples. Additionally, the weighted average over all measurements is shown as black dots, the dashed line connects these dots. The additions '_1to1' and '_1to2' denote the dilutions of the respective stock samples.
